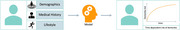# Machine learning model for early dementia risk prediction using common risk factors in primary care settings

**DOI:** 10.1002/alz70858_103344

**Published:** 2025-12-26

**Authors:** Jonas Botz, Anna‐Katharine Brem, Dag Aarsland, Holger Fröhlich, PREDICTOM Consortium

**Affiliations:** ^1^ Fraunhofer SCAI, Sankt Augustin, NRW, Germany; ^2^ University Hospital of Old Age Psychiatry, University of Bern, Bern, Switzerland; ^3^ King's College London, London, United Kingdom; ^4^ UK Dementia Research Institute, Institute of Psychiatry, Psychology & Neuroscience, King's College London., London, United Kingdom; ^5^ Centre for Age‐Related Medicine, Stavanger University Hospital, Stavanger, Stavanger, Norway; ^6^ Fraunhofer Institute for Algorithms and Scientific Computing SCAI, Sankt Augustin, Germany; ^7^ Bonn‐Aachen International Center for IT (b‐it), Bonn, Germany

## Abstract

**Background:**

Dementia poses an increasingly significant global health challenge, with projections indicating a dramatic rise in the number of affected individuals over the coming decades. This growing prevalence highlights the urgent need for early detection and intervention strategies. The 2024 Lancet Commission on dementia prevention, intervention, and care identified key risk factors for dementia, emphasizing the importance of early identification of high‐risk individuals to facilitate timely interventions that could potentially alter the disease's trajectory. These common risk factors can typically be gathered during routine general practitioner (GP) visits and do not necessitate specialized testing.

**Method:**

We developed and compared various machine learning models, including multi‐state‐models, random survival forest, CatBoost, and DeepHit, to predict the age dependent risk of a future dementia diagnosis (Figure 1). Our models were adjusted for the competing risk of deaths and trained on retrospective data from UK biobank, with external validation on data from the HUNT population study. All models incorporated common dementia risk factors as recommended by the Lancet Commission and were benchmarked against the previously published LIBRA score.

**Result:**

Our preliminary findings suggest that machine learning‐based risk models can accurately predict the likelihood of a future dementia diagnosis, achieving a 10‐fold cross‐validated area under the receiver operating characteristic curve (AUC) of 0.785 and 0.776 after 3 and 5 years, respectively.

**Conclusion:**

The models we developed can function as effective screening tools for estimating the risk of developing dementia 3 to 5 years in the future, utilizing easily accessible features at the GP level. The strength of these models lies in their incorporation of common risk factors that are routinely collected or readily available during standard GP visits. This approach represents a practical and scalable method for the early detection and prevention of dementia. By leveraging readily available data, these models could serve as valuable resources in primary care settings, potentially alleviating the global burden of dementia through timely interventions and personalized risk management strategies.

**Acknowledgment**

This Project Is Supported By The Innovative Health Initiative Joint Undertaking (IHIJU) Under Grant Agreement No 101132356. The JU Receives Support From The European Union's Horizon Europe Research And Innovation Programme. This Work Was Funded By UK Research AndInnovation (UKRI) Under The UK Government's Horizon Europe Funding Guarantee [UKRI Reference Number: 10083181]. In Switzerland The University Of Geneva Is Funded For PREDICTOM By The Swiss State Secretariat For Education Research And Innovation (SERI‐ Ref‐1131 52304).